# The Combination Strategy of Transarterial Chemoembolization and Radiofrequency Ablation or Microwave Ablation against Hepatocellular Carcinoma

**DOI:** 10.1155/2019/8619096

**Published:** 2019-08-26

**Authors:** Zhentian Xu, Haiyang Xie, Lin Zhou, Xinhua Chen, Shusen Zheng

**Affiliations:** ^1^Division of Hepatobiliary and Pancreatic Surgery, Department of Surgery First Affiliated Hospital, School of Medicine, Zhejiang University, NHFPC Key Laboratory of Combined Multi-Organ Transplantation, Key Laboratory of the Diagnosis and Treatment of Organ Transplantation, CAMS, Key Laboratory of Organ Transplantation, Zhejiang Province, Hangzhou 310003, China; ^2^Collaborative Innovation Center for Diagnosis Treatment of Infectious Diseases, China

## Abstract

Hepatocellular carcinoma (HCC) is the most common primary cancer of the liver. Hepatectomy and liver transplantation (LT) are regarded as the radical treatment, but great majority of patients are already in advanced stage on the first diagnosis and lose the surgery opportunity. Multifarious image-guided interventional therapies, termed as locoregional ablations, are recommended by various HCC guidelines for the clinical practice. Transarterial chemoembolization (TACE) is firstly recommended for intermediate-stage (Barcelona Clinic Liver Cancer (BCLC) B class) HCC but has lower necrosis rates. Radiofrequency ablation (RFA) is effective in treating HCCs smaller than 3 cm in size. Microwave ablation (MWA) can ablate larger tumor within a shorter time. Combination of TACE with RFA or MWA is effective and promising in treating larger HCC lesions but needs more clinical data to confirm its long-term outcome. The combination of TACE and RFA or MWA against hepatocellular carcinoma needs more clinical data for a better strategy. The characters and advantages of TACE, RFA, MWA, and TACE combined with RFA or MWA are reviewed to provide physician a better background on decision.

## 1. Introduction

Liver cancer is estimated to be ranked sixth on most currently diagnosed cancer as well as the fourth main reason of cancer death with about 841,000 new cases and 782,000 deaths occurred in 2018 worldwide [1]. Hepatocellular carcinoma (HCC) is the most common type of primary liver neoplasm and also one of the most common malignant tumors in the world [2, 3].

Surgeries including hepatectomy along with liver transplantation are curative potential treatments [4]. Unfortunately, less than 20% of patients are appropriate candidates for surgical resection and liver transplantation [4]. Systemic chemotherapy has not revealed beneficial on the survival rates of advanced HCC in the event of no valid treatment options until sorafenib was used as the targeted molecular remedy [5].

**Figure 1:**
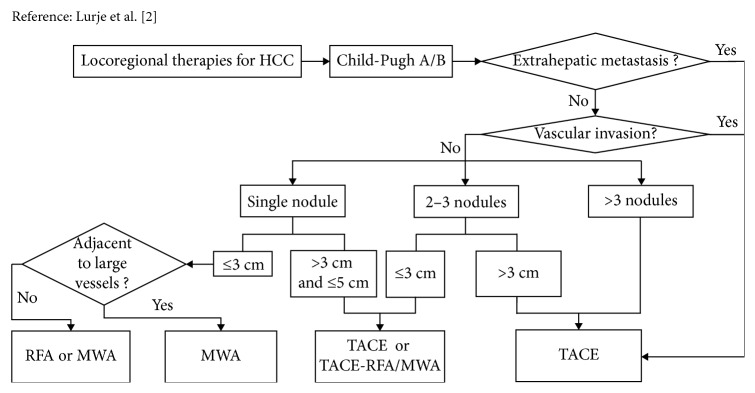
Locoregional therapies for HCC.

Locoregional therapies include transarterial chemoembolization (TACE), percutaneous ethanol injection (PEI), radiofrequency ablation (RFA), microwave ablation (MWA), cryoablation (CA), laser ablation, high-intensity focused ultrasound (HIFU), and irreversible electroporation (IRE) [2, 6]. Multifarious image-guided interventions now play a key role in treating HCC [7]. TACE is recommended as the first-line therapy for BCLC stage B HCC based on the Barcelona Clinic Liver Cancer (BCLC) guidelines. However, the necrosis rate of tumor cells is low and the intrahepatic recurrence rate of HCC is high using TACE alone [8]. Percutaneous thermal ablation is regarded as the optimum locoregional therapy choice for focal unresectable early-stage HCC [7]. Radiofrequency ablation and microwave ablation are important two types of ablative treatments. Furthermore, researchers have revealed that combined therapy was an effective selection on the therapy of patients with early or intermediate HCC at the moment of resection not being viable [9]. In this article, the profiles of TACE, RFA, MWA, and combination of TACE with RFA or MWA are reviewed based on the clinical data. Moreover, we provide some suggestions for locoregional therapies for HCC in Figure 1 on the basis of clinical data.

## 2. Transarterial Chemoembolization

Transarterial chemoembolization (TACE) is one kind of the arterially directed treatment methods currently besides transarterial embolization (TAE) and TACE with drug-eluting beads (DEB-TACE) [10]. It is the first-line applied therapy for patients with HCC in intermediate stage including unresectable, large, or multiple focal nodules without vascular involvement or extrahepatic metastasis [11]. TACE is confirmed effective by clinical trials and a meta-analysis [12]. Camma et al. [13] revealed that the overall 2-year mortality rate was obviously reduced in the TACE group than in the untreated group (OR, 0.54; 95% CI: 0.33, 0.89; *P* = 0.015) in a meta-analysis of 18 RCTs. TACE, a standard minimally invasive therapy, is aimed at delivering specific chemical with lipiodol mainly into the tumor area to result in necrosis and controlling the growth of tumor cells and to reduce the toxicity of chemotherapy of normal tissues [14]. The common regimens of TACE are cisplatin, mitomycin, doxorubicin, and epirubicin [15, 16]. The investigation conducted by Liu et al. [17] found that combination of chemotherapeutic regimens might improve survival rates as well as tumor response rates; gemcitabine seemed to be helpful to ameliorate the prognosis of HCC patients. However, at the moment of causing necrosis of tumor tissues by TACE, angiogenic factors like EGF and insulin-like growth factor 2 also increase; antiangiogenic drugs may be suggested in TACE-treated HCC [18].

Doxorubicin-eluting bead TACE (DEB-TACE) is a newly developed method based on conventional TACE (cTACE). A meta-analysis of seven studies (693 patients in total) compared DEB-TACE with cTACE [19]. It discovered that the pooled estimates for tumor response of DEB-TACE showed no difference compared with cTACE. Therefore, it indicated that DEB-TACE accomplishes the same as cTACE in tumor response. Interestingly, Zou et al. [20] concluded that DEB-TACE was superior to cTACE for higher complete response rates and overall survival rates for HCC patients.

As we have mentioned above, TACE used only leads to a low necrosis rate but a high intrahepatic recurrence rate of HCC. TACE can increase the risk of liver function failure especially in patients with Child-Pugh B cirrhosis because it can damage the liver parenchyma and the hepatic artery. Thus, Child-Pugh C liver function is mainly regarded as a contraindication for TACE [21, 22].

## 3. Radiofrequency Ablation

Radiofrequency ablation (RFA) was firstly applied for HCC patient in 1993 based on electromagnetic energy [23, 24]. An electrical current within the radiofrequency range is released through a needle electrode guided by imaging methods resulting in heat-based thermal cytotoxicity in RFA [25]. The creation and completion of an integrated electrical circuit are by means of finding the ground, generally a foil pad adhered to the thighs or back of patients [24]. Resistance encircling the electrodes produces heat with the temperatures ranging between 60°C and 100°C; the heat can cause almost instantaneous coagulation necrosis [24]. HCC tends to occur in the cirrhotic liver and often has its pseudocapsule; the cirrhotic liver along with pseudocapsule can serve as thermal insulators that lead to higher peak temperatures and prolong the time of cytotoxic temperatures. This is the so-called “oven effect” that makes RFA better efficiency in HCC than in hepatic metastases [26].

Usually, RFA can eliminate nodules no more than 3 cm in size, but if larger than 4 cm, it is not considered much effective [27]. In RFA, a solitary inserted electrode can cause necrosis of an area with the diameter equal to or less than 3.0 cm therefore ablating a 2 cm tumor completely [28]. A 0.5-1.0 cm safety margin of nontumor liver tissue is ablated to make sure that not only the peripheral tumor but also any microscopic extension are included [29]. According to the analysis conducted by Livraghi et al. [28], a complete necrosis of lesions up to 2 cm was achieved 90% with a locoregional recurrence rate of 1% and the estimable 3-year and 5-year survival rates were 76% and 55%, respectively, whereas another trial conducted by Livraghi et al. [30] included 80 HCCs with the tumors 3.1-5 cm in diameter (medium-sized tumors) and 46 HCCs with the tumors 5.1-9.5 cm in diameter (large-sized tumors) found that the complete necrosis (defined as 100% necrosis) was 61% in the medium-sized tumor group and 24% in the large-sized tumor group (*P* = 0.001). It reveals that RFA is perhaps an effective method in treating HCC lesions 3.1 cm or larger in diameter.

The efficacy of RFA is confined due to the diameter and location of tumor. RFA may cause inadequate ablation of perivascular tumor tissues because of the “heat-sink effect.” It is a phenomenon occurring as the energy disperses from the target lesion because of the blood flow. Thus, these tumor nodules near large vessels (>3 mm) should take modified treatment strategies to increase the success rate of therapy [31].

## 4. Microwave Ablation

Microwave ablation (MWA) is another type of ablation methodology using electromagnetic energy [24]. It was originated in the 1980s and 1990s [32]. MWA has become increasingly popular for its low cost and high ablation rate [33]. The high frequency electromagnetic energy (>900 MHz, generally 2450 MHz) is applied in MWA, leading dipole molecules, mainly water molecules, to continuous rotation in the oscillating electric field of microwave [34]. The drastic motion of dipoles produce frictional heat and cause coagulation necrosis in the target ablation zone [35].

MWA has several theoretical advantages in contrast of RFA. MWA can be applied for treating HCC in the patients with materials such as pacemaker or surgical clips in the body because complete electrical circuit is not requisite and grounding pads are not necessary [4]. Microwaves can reach a higher temperature in a shorter time and can generate a larger ablation area; MWA allows synergistic tissue heating of large or multifocal tumors because the machine can activate multiple antennae simultaneously [36]. Shorter treating time reducing the pain for patients is thought to be beneficial [37]. In addition, the heat-sink effect is attenuated, making MWA feasible in ablating the tumors that are adjacent to large vessels [37].

With the improvement of antennae and therapy strategies, MWA expands the ablation zone and can treat tumor of 5-8 cm in diameter [38]. MWA is now regarded as a curative treatment for the patients with very early stage HCC defined by the BCLC stage system with limited metastases. MWA is also a palliative therapy for HCC patients in BCLC B or C stage or inappropriate for other methods [38]. A multicenter study from China reported that 1-, 3-, and 5-year survival rates of 1007 patients with primary hepatic cancer treated by MWA were 91.2%, 72.5%, and 59.8%, respectively [39]. Another study conducted by Dong et al. [40] analyzed 234 HCC patients treated by MWA (mean tumor size, 4.1 ± 1.9 *cm*) and found that the 1-, 3-, and 5-year cumulative survival rates of patients were 92.70%, 72.85%, and 56.70%, respectively.

However, MWA may cause thermal injury [4]. Multiple antennae activated simultaneously may increase the range of treating zone whereas the interantenna distance may not be wholly covered and lead to incomplete ablation of the large tumor [4]. And a defect of MWA is high local development of tumor which may be caused by a larger applicator (5 mm in diameter) applied for tumor puncture increasing the risk of bleeding and subsequent tumor seeding [41].

## 5. Combination of Transarterial Chemoembolization and Radiofrequency Ablation

As mentioned above, RFA is feasible for small HCC because of its high complete ablation rate, but it is not recommended for larger lesions. Lesions adjacent to a large vessel (>3 mm) may not perform a complete necrosis owing to the so-called “heat-sink effect” [31]. Lessening or dispelling blood flow to restrain heat loss was confirmed to be capable of increasing the ablation volume [42]. In most studies, TACE has only achieved the complete necrosis rate of 10%-20% with the 1-, 3-, and 5-year overall survival rates at 49%-71.9%, 23%-62.5%, and 9%-17% [43]. Both of them have their own limitations. TACE followed by RFA has been more widely applied in recent years. The heat-sink effect of blood flow is reduced by lessening liver arterial flow after TACE procedure; meanwhile, the necrotizing effect of RFA treatment is increased in a tumor level. In addition, the zone of tumor necrosis in the treatment process of RFA is anticipated to be enlarged for the reason that ischemia and inflammation after TACE inducing the oedematous change [9].

Current clinical data reveal that TACE combed with RFA is superior to the single use of RFA or TACE alone in inducing higher complete necrosis and increasing overall survival rates [9]. The study conducted by Liu et al. [44] divided 88 patients into two groups (TACE group, TACE-RFA group); they found that the complete necrosis rates (CR) of the single TACE group and the TACE-RFA group were 27.9% (12/43) and 83.2% (37/45), respectively. Cao et al. [45] found that TACE-RFA was better than TACE used alone in 1-, 2-, and 3-year overall survival rates (OR_1‐*year*_ = 3.98, 95% CI: 2.87-5.51, *P* < 0.00001; OR_2‐*year*_ = 3.03, 95% CI: 2.10-4.38, *P* < 0.00001; OR_3‐*year*_ = 7.02, 95% CI: 4.14-11.92, *P* < 0.00001). A meta-analysis conducted by Ni et al. [43] suggested that combination of RFA and TACE had apparently higher overall survival rates and recurrence-free survival rates than RFA alone. Furthermore, Peng et al. [46] found that TACE-RFA treatment is superior to RFA used alone in overall survival and recurrence-free survival. TACE combined with RFA is considered a secure and efficient choice treating HCC patients despite not all the studies draw the same conclusion. However, TACE combined with RFA has no advantage for small lesions less than 3 cm, perhaps for the reason that RFA can reach complete necrosis alone making the TACE adding to RFA a superfluous way [9].

## 6. Combination of Transarterial Chemoembolization and Microwave Ablation

MWA has the advantage over RFA in ablating larger HCC lesions; nevertheless, it is also affected by the cooling effect more or less. Just like combining with RFA, TACE has its special superiority in attenuating heat loss by convection and leading to tissue necrosis and inflammatory edema by reducing local blood supply of tumor lesion [47, 48]. TACE selectively deliver the chemotherapeutics to targeted tumor, and the precaution of ischemic necrosis of the rest liver is realized [49]. Many factors confine the applying of TACE like size of tumors, incomplete ability eliminating tumor cells, local recurrence, and distant metastasis of remaining viable HCC cells [50].

Combination of TACE and MWA is another popular choice of interventional therapy and is confirmed effective. Many studies adopt MWA performed 2-4 weeks after TACE [50, 51]. Chen et al. [51] analyzed the data of 244 patients with HCC treated by TACE-MWA or TACE alone and found that the complete ablative rate in the TACE-MWA group was 92.1% and the TACE only group was 46.3% (*P* < 0.001), and they concluded that TACE-MWA led to better responses for HCC *tumors* ≤ 5 *cm* compared with the TACE group. Liu et al. [50] came into a conclusion that combination of MWA and TACE seemed to be a valid and potential modality in treating larger unresectable hepatocellular carcinoma based on their study. They chose 34 consecutive patients with large unresectable HCCs (>5 cm) and divided them into the TACE group and the TACE-MWA group. The reduction in tumor size was 61.7%, and the survival rate in the TACE-MWA group was observably higher than the TACE group (*P* < 0.003). A retrospective study conducted by Zheng et al. [52] involves 258 patients with a large solitary nodule or multinodular HCCs (≤10 nodules). They were treated by TACE-MWA (*n* = 92) or TACE alone (*n* = 166). The 1-, 2-, and 3-year overall survival (OS) rates were 85.9%, 59.8%, and 32.6% in the TACE-MWA group and 59.0%, 40.4%, and 11.4% in the TACE group, respectively (*P* < 0.001). The corresponding recurrence rates were 47.8%, 78.3%, and 94.6% in the TACE-MWA group and 74.7%, 96.4%, and 97.6% of that in the TACE group, respectively (*P* < 0.001).

## 7. Conclusion

Interventional therapies are appealing and confirmed to be beneficial for patients with HCCs. TACE combines with RFA or MWA is a better choice because of the specialty of TACE in reducing or preventing blood flow. As shown in Table 1, RFA and MWA present their advantages. RFA ablates HCC nodules in small sizes with lower local recurrence rates. Meanwhile, MWA does better on ablating whether small or large nodules but has higher local recurrence rates than RFA. Combination of RFA and TACE makes up the drawbacks using RFA alone. Many studies also reveal the efficacy of MWA combined with TACE, but more clinical data should be analyzed. Preliminary data in Table 2 has told us that combination therapy tend to be more effective than monotherapy. The study conducted by Abdelaziz et al. [49] showed that TACE-MWA tended to be higher complete response rates than TACE-RFA compared with TACE-RFA (*P* = 0.06) and resulted in better complete response rates with lesions 3-5 cm (*P* = 0.01) but had no difference in survival rates in treating HCC tumors.

## 8. Summary

RFA and MWA play a critical role for HCC. It is worth mentioning that TACE combined with either RFA or MWA is effective and promising in treating larger HCC lesions as preliminary data have proved. More clinical data need to be well analyzed to provide clinician better strategies in treating HCC.

## Figures and Tables

**Table 1:** Comparison of clinical studies in patients with HCC for radiofrequency ablation or microwave ablation.

References	Methods	Patients	Lesions	Mean age (years)	Size (cm)	Complete ablation rates (%)	Local recurrence rates (%)	Overall survival rates		
								1 yr (%)	3 yr (%)	5 yr (%)
Livraghi et al. [28]	RFA	218	—	68	≤2.0	98.1	0.9	—	76	55
Livraghi et al. [30]	RFA	114	126	64.4	5.4 (mean)	47.6	—	—	—	—
Liang et al. [39]	MWA	1007	1363	56.3	1.0-18.5 2.1 ± 1.8 (mean)	97.1^a^	5.9	91.2	72.5	59.8
Dong et al. [40]	MWA	234	339	54.8 ± 11.4	1.2-8.0 4.1 ± 1.9 (mean)	92.0 (US)^b^	7.3	92.7^c^	72.85^c^	56.7^c^

^a^Technique effectiveness; ^b^color Doppler flow signals disappeared in 92.0% (263/286) of the lesions; ^c^cumulative survival rates.

**Table 2:** The efficacy of combination of TACE with RFA or MWA vs. monotherapy.

References	Methods	Patients	Age (years)	Size (cm)	Response rates (%)	Overall survival (OS) rates (%)				OS *P* value
						0.5 yr	1 yr	1.5 yr	2 yr	
Liu et al. [44]	TACE	43	44-78	5-14	67.4	—	—	—	—	0.081
	TACE-RFA	45	45-75	4-15	91.1	—	—	—	—	

Peng et al. [46]	RFA	95	55.3 ± 13.3	3.39 ± 1.35	96.8	—	66.6	—	—	0.002
	TACE-RFA	94	53.3 ± 11.0	3.47 ± 1.44	96.8	—	92.6	—	—	

Liu et al. [50]	TACE	18	51.9 ± 13.6	6.7 ± 1.5	38.9	50	11.1	0	0	0.003
	TACE-MWA	16	52.1 ± 14.5	6.8 ± 1.5	87.5	75	33.3	18.7	6.25	

Chen et al. [51]	TACE	96	59.7 ± 10.5	2.88 ± 1.25	46.3	96.9	87.2	81.1	77	0.317
	TACE-MWA	48	58.8 ± 9.6	2.74 ± 1.09	92.1	100	91.7	88.5	88.5	

Zheng et al. [52]	TACE	166	54.6 ± 10.5	8.5 ± 2.5	55.4	—	59	—	40.4	<0.001
	TACE-MWA	92	53.3 ± 8.2	9.1 ± 2.8	81.5	—	85.9	—	59.8	

## Abbreviations

BCLC: Barcelona Clinic Liver Cancer
CA: Cryoablation
cTACE: Conventional transarterial chemoembolization
DEB-TACE: Doxorubicin-eluting bead transarterial chemoembolization
HCC: Hepatocellular carcinoma
HIFU: High-intensity focused ultrasound
IRE: Irreversible electroporation
LT: Liver transplantation
MELD: Mayor model for end stage liver disease
MWA: Microwave ablation
RFA: Radiofrequency ablation
TACE: Transarterial chemoembolization
TAE: Transarterial embolization.
